# The expression and prognostic value of the guanine nucleotide exchange factors (GEFs) Trio, Vav1 and TIAM-1 in human breast cancer

**DOI:** 10.1186/1477-7800-5-23

**Published:** 2008-10-16

**Authors:** Jane Lane, Tracey A Martin, Robert E Mansel, Wen G Jiang

**Affiliations:** 1 Metastasis research Group, University Department of Surgery, Cardiff University School of Medicine, Heath Park, Cardiff, CF4 4XN, UK

## Abstract

**Background:**

Development of metastasis in breast cancer is a multi-step process comprising changes in cytoskeletal structure and gene expression of tumour cells leading to changes in cell adhesion and motility. The Rho GTPase proteins, which function as guanine nucleotide regulated binary switches, govern a variety of cellular processes including cell motility and migration, changes in cell adhesion as well as actin cytoskeletal reorganisation and gene expression/transcription. One group of activators which regulate the Rho-GTPases is the guanine nucleotide exchange factors (GEFs), and this study looked at three such GEFs, Trio, Vav1 and TIAM-1. The purpose of this study was to investigate the expression of these GEFs, in human breast cancer and assess the affect on clinical outcome.

**Methods:**

Specimens of fresh, frozen breast tumour tissue (n = 113) and normal background tissue (n = 30) were processed for quantitative PCR analysis. The expression and levels of expression of Trio, Vav1 and TIAM-1 were analysed using RT-PCR and real-time Q-PCR respectively. Sections were also immunostained with Trio and Tiam-1 antibodies.

**Results:**

Tumour tissue exhibited high levels of all three Rho activators Trio, Vav1 and TIAM-1 compared with normal background breast tissue, reaching a level of significance for the GEF Trio (p = 0.013). Trio levels also increased significantly in patients with a poor prognostic index (p = 0.04).

Levels of TIAM-1 were significantly higher in tumour tissue from patients who died from breast cancer compared with those who survived (p = 0.04). No significant correlation was found between tumour grade and histology types.

**Conclusion:**

High expression levels of Trio, Vav1 and TIAM-1 were seen in breast tumours, especially in those with poor prognosis. This suggests that aberrant regulation of Rho family activities by GEFs may have an important prognostic value in breast cancer.

## Background

During the development of metastasis in breast cancer, tumour cells undergo numerous changes in their cytoskeletal structure and gene expression promoting changes in cell adhesion, motility and morphology leading to metastasis and tissue invasion. The Rho GTPases, which function as guanine nucleotide regulated binary switches, control the regulation of the actin cytoskeleton, and as such, have been implicated in promoting a variety of cellular processes including cell motility and migration, changes in cell adhesion as well as actin cytoskeletal reorganisation [1-3] and gene expression/transcription [4]. Increased expression of Rho proteins has been demonstrated in a variety of tumours with raised levels of Rho-C, Rho-G and Rho-6 detected in breast tumour tissue [5], as well as increase in the expression of the ROCK proteins, which function as downstream effectors of the Rho GTPases [6]. Therefore this study was initiated to investigate the expression levels of the activators of the Rho-GTPase cycle.

In part, the Rho-GTPases are activated by guanine nucleotide exchange factors (GEFs), a group of regulators which function as modulators of the activation/inactivation cycle of the Rho family GTPases by binding to inactive GTPases and inducing a conformational change leading to GDP release. The GTPases then bind free cytoplasmic GTP to become reactivated.

There are a large number of guanine nucleotide-binding proteins requiring an equally large range of GEFs to ensure signalling specificity and, as such, a number of GEF families exist. A recent review of the GEFs and GAPs (GTPase activating proteins), which both function as regulators of the Rho GDP/GTP cycle, has suggested that these proteins may be potential therapeutic targets for developing drug treatments for various cancers [7]. Three such GEFs which regulate the Rho family of GTPases are Trio, Vav1 and TIAM-1 (T-lymphoma invasion and metastasis gene).

Trio acts as a cytoskeletal modulator activating the Rho and/or Rac pathways and has been shown to play a vital role in axon guidance, neuronal cell migration and cell motility [8] as well as in the regulation of focal adhesion dynamics [9].

The Vav family of guanine nucleotide exchange factors have been shown to modulate activity of Rho, Rac and/or Cdc42 to effect changes in cytoskeletal organisation [10].

Vav proteins couple tyrosine kinase signals with the activation of the Rho-GTPases and are likely to play an integral role in the regulation of cell differentiation in many tissues. Vav1 has been shown to function as an oncogene involved in malignant transformation. This protein also acts as a growth stimulatory protein in primary pancreatic adenocarcinoma. [11].

Studies have shown that over expression of TIAM-1 protein confers an invasive phenotype in T-lymphoma cells suggesting that increased TIAM-1 levels may lead to tumour progression and invasion [12]. This GEF has also been shown to interact with the cytoskeletal protein ankyrin which promotes Rac activation leading to breast tumour cell invasion and migration [13].

To look for evidence to support their role in the motility and invasion of breast tumour cells we have analysed the expression of the Rho GTPase regulators Trio, Vav1 and TIAM-1 in normal breast tissue and compared this with the expression in breast tumour tissue and with the grade of tumour and clinical outcome.

## Methods

Surgical specimens of fresh, frozen breast tissue comprising breast tumours (n = 113) and normal background tissue (n = 30) were collected during surgery. Information was available on the Nottingham Prognostic Index (NPI), grade of tumour, degree of nodal involvement and clinical outcome for all patients with a mean follow up period of 72.2 months. The expression and levels of expression of Trio, Vav1 and TIAM-1 were analysed using RT-PCR and real-time quantitative PCR respectively.

### RNA extraction and RT-PCR

RNA was isolated from breast cancer cell and tissue lines using a standard RNA-zol procedure, as we previously reported [6]. For RT-PCR, cDNA was synthesised in a 25 μl reaction mixture, as described in the manufacturer's protocol (ABgene Reverse Transcription System, ABgene, Surrey, UK). The cDNA obtained was amplified by a standard PCR mixture (as supplied in Pre-aliquoted Reddy-Load Mix, Advanced Biotechnologies). Cycling conditions for the 25 μl reaction mix were 94°C for 4 min, followed by 36 cycles of 94°C for 15 s, 55°C annealing for 15 s and 72°C for 30 s, followed by a final extension of 7 min at 72°C. The products were visualised on a 0.8% agarose gel following staining with ethidium bromide.

### Quantitative-PCR analysis

The Q-PCR system used the Amplifluor™ Uniprimer™ system (Intergen Company Oxford, UK) and Thermo-Start^® ^(ABgene, Epsom, Surrey, UK) [5,6]. Specific primers were designed by the authors and manufactured by Invitrogen (Invitrogen Life Technologies, Paisley, Scotland, UK). Using the Icycler IQ system (Bio-Rad, Hemel Hempstead, UK), which incorporates a gradient thermocycler and a 96-channel optical unit, the plasmid standards and breast cancer cell cDNA were simultaneously assayed in duplicated 10 μl reactions as follows: Q-master mix (5 μl), forward primer (0.3 μl), probe (0.3 μl), reverse primer (0.3 μl), plasmid, (internal standard) or specimen cDNA (4 μl), water (0.1 μl). Q-PCR conditions were as follows: enzyme activation at 95°C for 12 min, 1 cycle, followed by 60 cycles of denaturing: 95°C for 15 s; annealing: 55°C for 40 s; extension: 72°C for 25 s. Using purified plasmids as internal standards, the level of each molecule cDNA (copies/50 ng RNA) in the breast cancer samples was calculated. Q-PCR for β-actin was also performed on the same samples, to correct for any residual differences in the initial level of RNA in the specimens (in addition to spectrophotometry). The products of Q-PCR were verified on agarose gels. Primer pairs for Q-PCR were as follows: Trio F1 (5'-accgttgttcttagatgtcg); Trio ZR (5'-actgaacctgaccgtacaggagatgctgtagtgaccat; Vav1 F1 (5'-agtctctggacaccacctt); Vav1 ZR (5'-actgaacctgaccgtacaccaaaatactttgtgcttcc); TIAM-1 F1 (5'-ctttaagaagaaacgctccca); TIAM-1 ZR (5'-actgaacctgaccgtacacttggctcagatcagagagt).

### Immunohistochemistry

Frozen sections of breast tumour and background tissue were cut with a cryostat at a thickness of 6 μm. The sections were mounted on super frost plus microscope slides, air dried and then fixed in a mixture of 50% acetone and 50% methanol. The sections were then placed in "Optimax" wash buffer for 5 – 10 minutes to rehydrate and incubated for 20 mins in a 0.6% BSA blocking solution and probed with either Trio (sc-6060) or Tiam-1 (sc-872) primary antibody (Santa Cruz Biotechnologies Inc., CA, USA). Following extensive washings in buffer, sections were incubated for 30 min in the secondary biotinylated antibody (Multilink Swine anti-goat/mouse/rabbit immunoglobulin, Dako Inc.). Following washings, Avidin/Biotin Complex (Vector Laboratories) was applied to the sections followed by extensive washings. Diamino benzidine chromogen (Vector Labs) was then added to the sections which were incubated in the dark for 5 min. Sections were counterstained in Gill's Haematoxylin and dehydrated in ascending grades of methanol before clearing in xylene and mounting under a cover slip.

### Statistical analysis

Statistical analysis was performed with MINITAB version 11.2 (Minitab Inc., State College, PA) using two sample Student's *t*-tests.

## Results

### Aberrant expression of TRIO, VAV1 and TIAM-1 in human breast cancer

**Figure 1:**
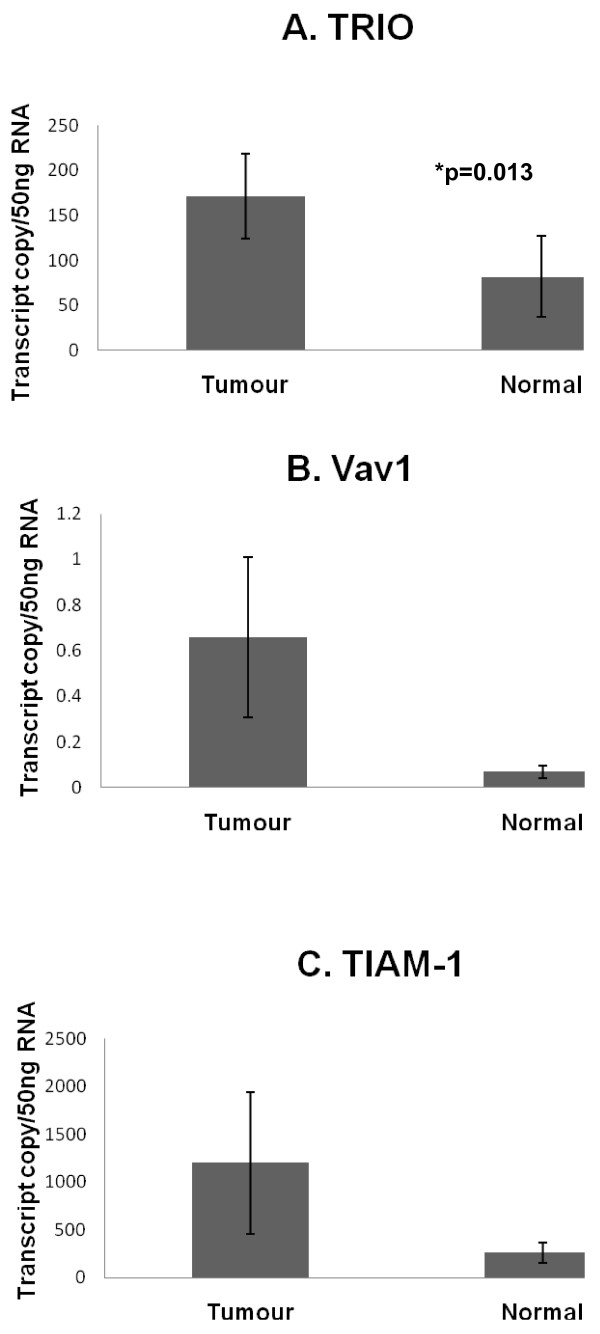
**Levels of expression of Trio, Vav1 and TIAM-1 in tumour and normal background breast tissue**. Comparison of levels of Trio, Vav1 and TIAM-1 in tumour samples compared with normal background tissue (expressed as transcript copy number per 50 μg of messenger RNA).

**Figure 2:**
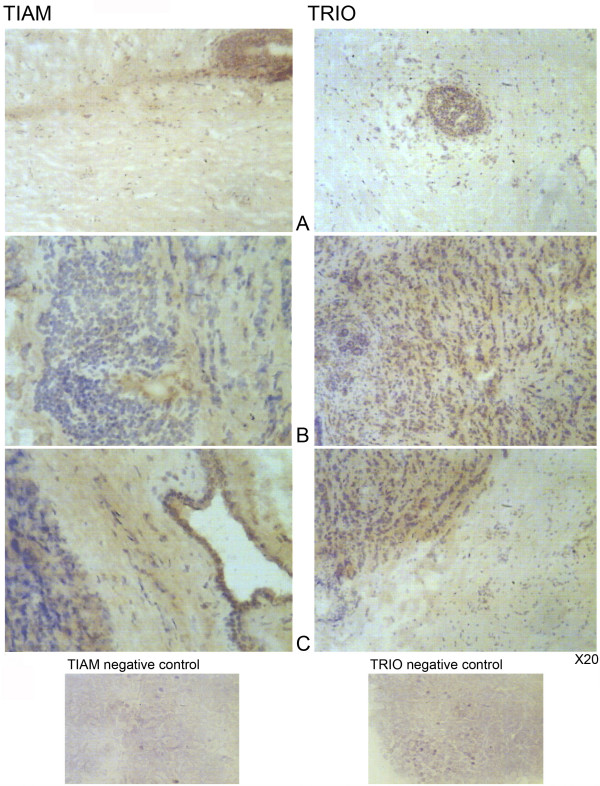
**Immunohistochemical staining of TIAM-1 and Trio in tumour and normal background breast tissue**. Staining of TIAM-1 (*left panel*) and Trio (*right panel*) in normal background tissue (A) and in breast tumour tissue (B). Distribution of staining of TIAM-1 and Trio in tumour and surrounding normal tissue (C).

Tumour tissue exhibited significantly high levels of Trio, when compared with normal background breast tissue (171.5 ± 46.7 v 82.0 ± 45; p = 0.013). Levels of Vav 1 and TIAM-1 were also higher in tumours but were not significantly different (0.66 ± 0.35 v 0.07 ± 0.027; p = 0.095 for Vav1 and 1196 ± 743 v 261 ± 107; p = 0.22 for TIAM-1) (Figure 1). These results were confirmed for Trio and TIAM-1 by immunohistochemistry staining (Figure 2); with very little staining seen in normal background tissue (Figure 2A) while tumour tissue stained strongly for both Trio and TIAM-1 (Figure 2B). This difference of staining intensity between normal and tumour tissue is shown in Figure 2C.

**Figure 3:**
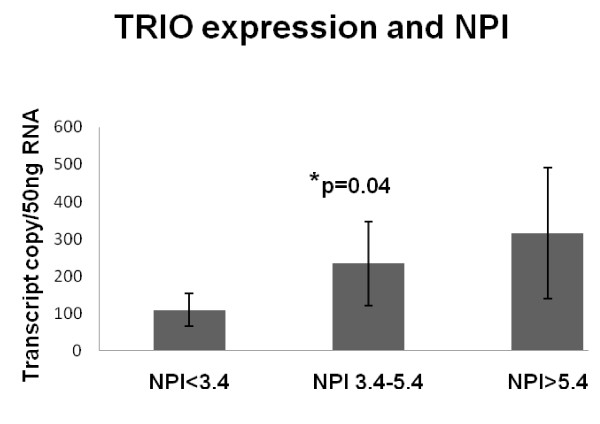
**Levels of expression of Trio in relation to NPI status**. Trio levels increased sequentially with an increase in the Nottingham Prognostic Index.

The expression levels of all three GEFs, assessed by Q-PCR, were compared with a series of predictive factors (Tables 1, 2, 3). Trio levels were seen to increase significantly with an increase in Nottingham Prognostic Index (109 ± 44 NPI1; 234 ± 113 NPI2; 315 ± 176 NPI3 p = 0.04 comparing NPI1 and NPI2) Figure 3.

**Table 1:** Expression levels of the GEFs studied related to NPI status of the tumours

	Guanine Nucleotide Exchange Factors		
NPI status	TRIO	VAV1	TIAM-1

(NPI < 3.4)	109 ± 44	0.45 ± 0.22	1957 ± 1466

(NPI 3.4–5.4)	234 ± 113	1.18 ± 1.06	506 ± 205

(NPI > 5.4)	315 ± 176	0.45 ± 0.41	298 ± 123

NPI = Nottingham Prognostic Index.

Levels of Trio showed a sequential increase with increased NPI status with a significant increase from NPI1 to NPI2 (p = 0.04). Expression of Vav1 showed no definitive pattern while TIAM-1 expression showed a sequential decrease with an increase in NPI status.

**Table 2:** Expression levels of the three GEFs studied related to tnm status

	Guanine Nucleotide Exchange Factors		
TNM status	TRIO	VAV1	TIAM-1

1	160 ± 50.5	0.41 ± 0.22	430 ± 117

2	116.5 ± 61.6	1.28 ± 1.04	2613 ± 2245

3	669 ± 544	0.148 ± 0.12	2022 ± 1817

tnm = tumour-node-metastasis.

TIAM-1 expression increased with tumour-node-metastasis (tnm) status as did Vav1 levels however, Trio expression showed an increase from tnm1 to tnm3 (160 ± 50 tnm1 and 669 ± 554 tnm4 p = 0.39) but a decrease in Trio expression in tnm2 tumours (117 ± 62).

**Table 3:** Expression levels of the three GEFs studied related to clinical outcome

	Guanine Nucleotide Exchange Factors		
Survival status	TRIO	VAV1	TIAM-1

1 Disease free	174.1 ± 62.1	0.89 ± 0.48	60.3

2 Metastatic disease	263 ± 151	0.01 ± 0.003	169

3 Local recurrance	152.4 ± 94.5	0.02 ± 0.012	26

4 Died from breast cancer	188.6 ± 97.2	0.03 ± 0.016	225

Trio levels show little change of expression between patients who survived and patients who died from breast cancer. Vav1expression was highest in patients who remained disease free while TIAM-1 expression was much lower in patients who remained disease free compared with those who died from breast cancer.

Expression of Vav1 showed no definitive pattern, increasing in NPI2 tumours compared with NPI1 tumours but showing no change in NPI3 tumours (mean ± SEM 0.45 ± 0.22 NPI1; 1.18 ± 1.06 NPI2; 0.45 ± 0.41 NPI3). TIAM-1 expression showed a sequential decrease with increase in NPI status (1957 ± 1466 NPI1; 506 ± 205 NPI2; 298 ± 123 NPI3) however these changes did not reach a level of significance (p > 0.3 in all cases) Table 1.

TIAM-1 expression increased with tumour-node-metastasis status (mean ± SEM 430 ± 117 TNM1 and 2613 ± 2245 TNM2 p = 0.34), as did Vav1 levels (mean ± SEM 0.407 ± 0.22 TNM 1 and 1.28 ± 1.04 TNM 2 p = 0.42) however, Trio expression showed an increase from TNM1 to TNM3 (160 ± 50 TNM1 and 669 ± 554 TNM4 p = 0.39) but a decrease in Trio expression in TNM2 tumours (117 ± 62) Table 2.

Expression levels of the three GEFs studied were also related to the follow-up data available on clinical outcome for the patients (Table 3). Based on the outcomes of the follow up data, patients were grouped into those who were disease free; with metastatic disease; with local recurrence of breast cancer, and died as a consequence of breast cancer. Trio levels showed little change of expression between those patients who survived and those who died from their breast cancer (mean ± SEM 174.1 ± 62.1 surv 1; 263 ± 151 surv 2; 152.4 ± 94.5 surv 3; 188.6 ± 97.2 surv 4). Vav1 showed the highest expression in patients who remained disease free (0.89 ± 0.48) with much lower expression in those patients with metastatic disease (0.01 ± 0.003); with local recurrence (0.02 ± 0.012) and in patients who died from breast cancer (0.03 ± 0.016). Levels of TIAM-1 were much lower in tumour tissue from patients who remained disease free compared with those who died from breast cancer (median value disease free, 60.3 v median value died from breast cancer, 224.5; p = 0.04, with range Q1–Q3 disease free 0 – -251.1; died from breast cancer 0 – -3.2).

Comparison of tumour types showed lower levels of Trio (p = 0.33) and TIAM-1 (p = 0.37) but higher levels of Vav1 (p = 0.47) in ductal tumours than in all other tumour types Table 4.

**Table 4:** Expression levels of the three GEFs studied related to type of breast cancer

	Guanine Nucleotide Exchange Factors		
Breast cancer type	TRIO	VAV1	TIAM-1

Ductal	130 ± 34	0.73 ± 0.44	487 ± 152

Other	578 ± 410	0.36 ± 0.24	12029 ± 11862

	P = 0.33	P = 0.47	P = 0.37

Lower levels of Trio and TIAM-1 were found in ductal tumours than in all other tumour types

## Discussion

The guanine nucleotide exchange factors TRIO, VAV1 and TIAM-1 activate the Rho-GTPases by initiating GDP/GTP exchange. The GEF Trio is associated with cell motility by regulation of FAK and effects changes in the actin cytoskeleton. This study has shown for the first time that Trio levels are significantly raised in breast tumour tissue compared with normal tissue (p = 0.013) and higher levels of Trio are also found in tumours with a poor predictive outcome (NPI > 5.4) (p = 0.04). This pattern is confirmed by immunohistochemical staining.

Vav1 functions as an oncogene involved in malignant transformation [14] and the Vav family appears to play an essential role in angiogenesis [15] and androgen receptor transcriptional activity in prostate cancer [16]. It is expressed in human neuroblastomas originating from tissues which do not normally express this protein [17]. The results of our study show that Vav 1 expression was higher in tumour tissue than in normal background tissue but this did not reach a level of significance (p = 0.095). Vav 1 over expression has been previously shown to be associated with poorer survival (9,10) however our study indicates that decreased levels of Vav 1 are associated with poorer survival (p = 0.07 in all cases).

Higher grade breast tumours have a higher expression of TIAM-1 which can disturb intercellular adhesiveness by inducing cellular ruffles and may be correlated with the invasive phenotypes of human breast tumours [18]. The results of our study would tend to agree with this finding as we have shown that TIAM-1 expression is higher in tumour tissue than in normal background breast tissue, although this did not reach a level of significance (p = 0.22). The results from immunohistochemical staining agree with this finding. However when looking at clinical outcome for these patients, levels of TIAM-1 were significantly higher in tumour tissue from patients who died from breast cancer compared with those who survived p = 0.04.

## Conclusion

High levels of expression of all three GEFs studied Trio, Vav1 and TIAM-1 were seen in breast tumours compared with normal background breast tissue. The statistical evidence in this study leads to the conclusion that the GEF TRIO may be particularly useful as a prognostic factor in breast cancer as its level is significantly increased in tumour tissue with Tiam-1 showing a significant increase in tumour tissue from patients with poor prognosis. This suggests that aberrant regulation of Rho family activities by GEFs may have an important prognostic value in breast cancer and that these GEFs may be important targets for therapeutic intervention.

## Competing interests

The authors declare that they have no competing interests.

## Authors' contributions

JL carried out the RNA extraction, PCR and Q-PCR analysis and drafted the manuscript. TM carried out tissue preparation and PCR analysis and helped with drafting the manuscript and statistical analysis. REM provided the patient samples for this study. WGJ conceived the study, participated in its design and helped to draft the manuscript. All authors have read and approved the final manuscript.

## References

[B1] Hall A (1998). Rho GTPases and the actin cytoskeleton. Science.

[B2] Ridley AJ (2001). Rho GTPases and cell migration. J Cell Sci.

[B3] Takaishi K, Sasaki T, Kotani H, Nishioka H, Takai Y (1997). Regulation of cell-cell adhesion by rac and rho small G proteins in MDCK cells. J Cell Biol.

[B4] Jaffe AB, Hall A (2002). Rho GTPases in transformation and metastasis. Adv Cancer Res.

[B5] Jiang WG, Watkins G, Lane J, Cunnick GH, Douglas-Jones A, Mokbel K, Mansel RE (2003). Prognostic value of Rho-GTPases and Rho Guanine Nucleotide Dissociation Inhibitors (GDI's) in human breast cancers. Clin Cancer Res.

[B6] Lane J, Martin TA, Watkins G, Mansel RE, Jiang WG (2008). The expression and prognostic value of ROCK I and ROCK II and their role in human breast cancer. Int J Oncol.

[B7] Bos Jl, Rehmann H, Wittinghofer A (2007). GEFs and GAPs: Critical elements in the control of small G proteins. Cell.

[B8] Bateman J, van Vactor D (2001). The Trio family of guanine-nucleotide exchange factors: regulators of axon guidance. J Cell Science.

[B9] Medley QG, Buchbinder EG, Tachibana K, Ngo H, Serra-Pages C, Streuli M (2003). Signalling between focal adhesion kinase and Trio. J Biol Chem.

[B10] Hornstein I, Alcover A, Katzav S (2004). Vav proteins, masters of the world of cytoskeletal organisation. Cell Signal.

[B11] Fernandez-Zapico ME, Gonzalez-Paz NC, Weiss E, Savoy DN, Molina JR, Fonseca R, Smyrk TC, Chari ST, Urrutia R, Billadeau DD (2005). Ectopic expression of VAV1 reveals an unexpected role in pancreatic cancer tumorigenesis. Cancer Cell.

[B12] Habets GGM, Schlotes EHM, Zuydgeest D, Kamma RA van der, Stam JC, Berns A, Collard JG (1994). Identification of an invasion inducing gene Tiam-1, that encodes a protein with homology to GDP-GTP exchangers for Rho-like proteins. Cell.

[B13] Borguignon LY, Zhu H, Shao L, Chen YW (2000). Ankyrin-Tiam1 interaction promotes Rac1 signaling and metastatic breast tumor cell invasion and migration. J Cell Biol.

[B14] Bustelo XR (2000). Regulatory and signalling properties of the Vav family. Mol Cell Biol.

[B15] Hunter SG, Zhuang G, Brantley-Sieders D, Swat W, Cowan CW, Chen J (2006). Essential role of Vav family guanine nucleotide exchange factors in EphA receptor-mediated angiogenesis. Mol Cell Biol.

[B16] Lyons LS, Burnstein KL (2006). Vav3, a Rho GTPase guanine nucleotide exchange factor, increases during progression to androgen independence in prostate cancer cells and potentiates androgen receptor transcriptional activity. Mol Endocrinol.

[B17] Hornstein I, Pikarsky E, Groysman M, Amir G, Peylan-Ramu N, Shulamit K (2003). The haematopoietic specific signal transducer Vav1 is expressed in a subset of human neuroblastomas. J Pathol.

[B18] Adam L, Vadlamudi RK, McCrea P, Kumar R (2001). Tiam1 overexpression potentiates heregulin-induced lymphoid enhancer factor-1/beta-catenin nuclear signaling in breast cancer cells by modulating the intercellular stability. J Biol Chem.

